# Focal non granulomatous orchitis in a patient with Crohn’s disease

**DOI:** 10.1186/s13000-015-0273-5

**Published:** 2015-04-28

**Authors:** Nicolas Piton, Marie-Laurence Roquet, Louis Sibert, Jean-Christophe Sabourin

**Affiliations:** Department of Pathology, Rouen University Hospital, Rouen, France; Department of Urology, Rouen University Hospital, Rouen, France

**Keywords:** Non-granulomatous orchitis, Focal orchitis, Crohn’s disease, Orchiectomy

## Abstract

Crohn’s disease is a systemic disease and sometimes involves the testicle, usually leading to granulomatous lesions. We report herein a case of focal non-granulomatous orchitis in a 21-year-old patient with active Crohn’s disease treated by an anti-tumor necrosis factor monoclonal antibody. This circumscribed testicular lesion mimicked a tumor, leading to orchiectomy. Pre-operative blood tests (i.e. alpha-fetoprotein, lactate dehydrogenase and human chorionic gonadotrophin) were strictly normal Pathological examination of the testicle revealed a focal inflammatory infiltrate predominantly composed of lymphocytes accompanied by few plasma cells, lacking giant cells or granulomas. Importantly, intratubular germ cell neoplasia, atrophy or lithiasis were not observed.

After discussing and excluding other plausible causes (burnt-out /regressed germ cell tumor, infection, vascular or traumatic lesions, iatrogenic effects), we concluded that this particular case of orchitis was most likely an extra-digestive manifestation of inflammatory bowel disease. To our knowledge, this is the first described case of focal non-granulomatous orchitis associated with Crohn’s disease.

**Virtual Slides:** The virtual slide(s) for this article can be found here: http://www.diagnosticpathology.diagnomx.eu/vs/2117747284160112

## Background

Crohn’s disease is a disease mainly involving the gut. However, it is estimated that at least 25% of patients will have some extra-digestive symptoms during the natural history of their condition. Organ systems involved are mainly mucocutaneous (erythema nodosum, pyoderma gangrenosum, aphtous ulceration granulomatous and multinucleated giant cell lesions), musculoskeletal (peripheral joint inflammation, axial arthritis, osteoporosis) and ocular (episcleritis, uveitis, hyperhemia, visual deficits) [1].

The testicle is very rarely impacted, usually leading to granulomatous lesions [2]. We report herein a case of what we believe to be non granulomatous orchitis related to Crohn’s disease in a 21-year-old patient.

### Case presentation

A 21-year-old man presented with chronic right testicular pain which appeared between 1 and 2 years before he consulted. He had a medical history of Crohn’s disease of the small bowel, successfully treated by monoclonal antibody infliximab (5 mg/kg every 9 weeks). No extra-intestinal manifestation, before or after the therapy, had been noticed prior to this genital pain. Clinically, the testicles were of normal volume and consistency. Examination by palpation was painless and did not retrieve any nodule. Ultrasound examination revealed a heterogeneous and hypoechoic lesion measuring 2 cm in length and 0.7 cm in width at the upper portion of the right testicle. Doppler examination showed that the lesion was hypervascularized. An injected scan of the chest, abdomen and pelvis revealed several lymph nodes in the mediastinum less than 1 cm in size, accompanied by centimetric mesenteric lymph nodes. Given the suspicion of malignancy, right orchiectomy was decided. Pre-operative blood tests were strictly normal, such as alpha-fetoprotein (142 IU/L), lactate dehydrogenase (142 IU/L) or human chorionic gonadotrophin (<1 IU/L).

After formalin fixation, the specimen consisted of a 6-cm long testicle, weighing 53 grams. The spermatic cord was 7 cm long. The cut surface showed a peripheral cuneiform lesion measuring 15 mm long, darker in color than the rest of the testis. The whole lesion was submitted for histological examination. Histological samples of macroscopically normal testicle were also performed.

**Figure 1:**
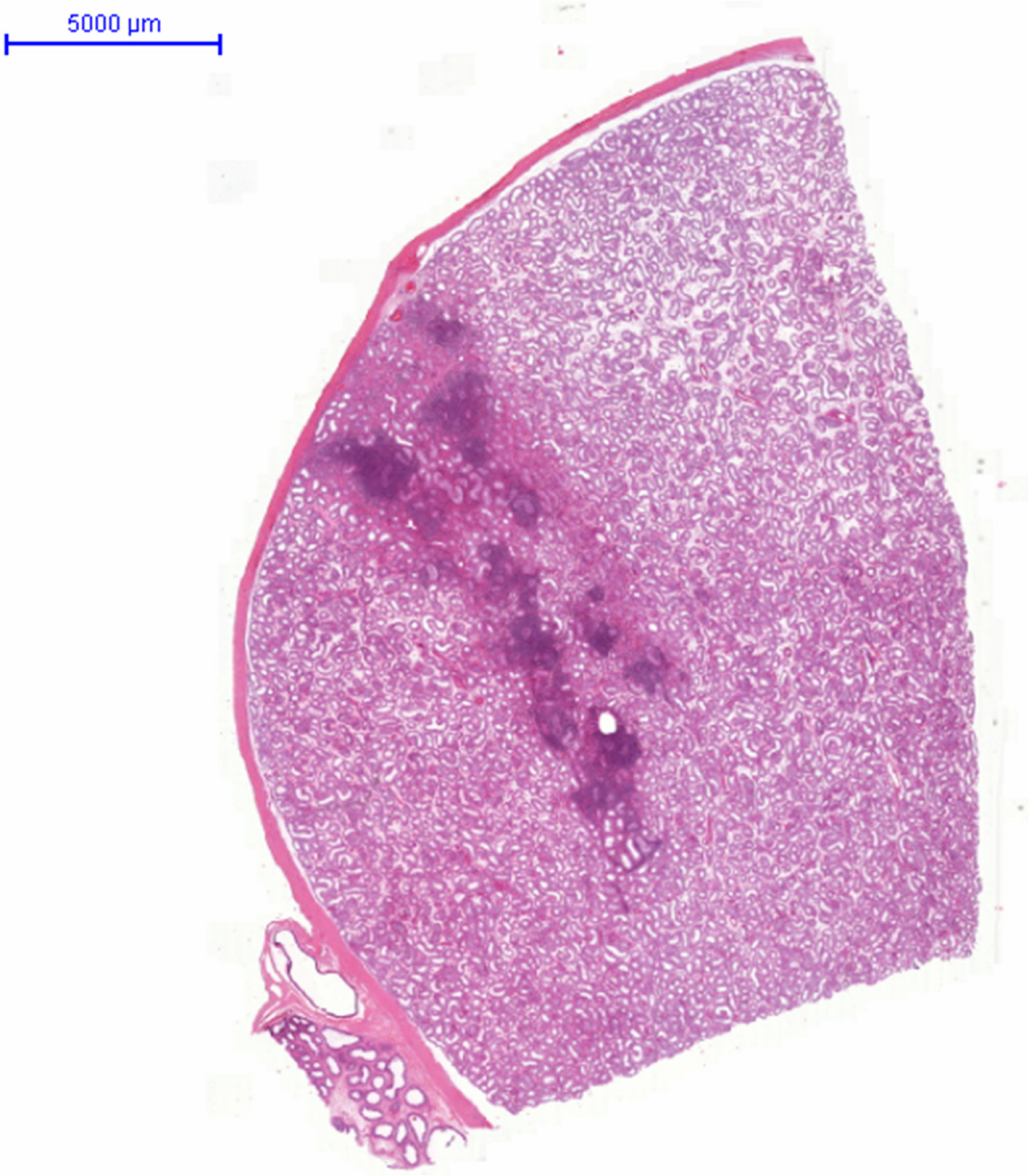
Photomicrograph of tissue section after staining by Hematoxylin Eosin and Safran, showing a focal cuneiform inflammatory infiltrate. The bar scale indicates 5000 μm.

**Figure 2:**
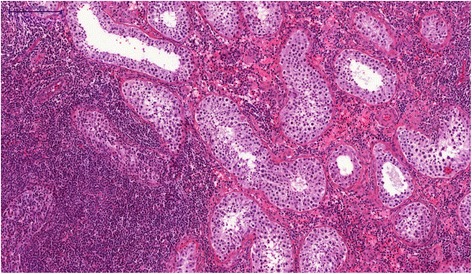
Photomicrograph of tissue section after staining by Hematoxylin, Eosin and Safran. The inflammatory infiltrate is composed of small lymphocytes accompanied by few plasma cells, and is mainly around the tubules, with some images of exocytosis. No giant cell or granulomatous lesion was observed. The bar scale indicates 200 μm.

**Figure 3:**
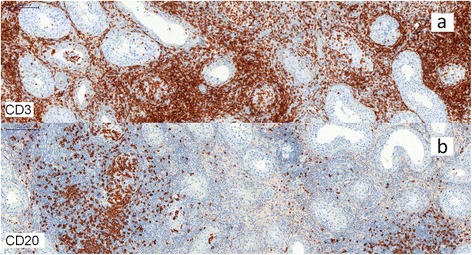
Photomicrographs of tissue section immunostained with anti-CD3 antibody (Figure 3a) and anti-CD20 antibody (Figure 3b), showing a majority of T cells (CD3 +) with only a few B cells (CD20 +). The bar scale indicates 200 μm.

Histologically, Hematoxylin, Eosin and Safran staining revealed a chronic inflammatory infiltrate consisting predominantly of small lymphocytes accompanied by few plasma cells. These inflammatory cells were mainly located around the seminiferous tubules and some of these elements in exocytosis were observed in the tubules (Figures 1 and 2). There were very few histiocytes within the tubules. In some territories, the lamina propria was predominantly edematous, without inflammatory cells. Neither giant cells nor granulomas were present. No vascular lesion was observed. Inflammation was strictly absent in the rest of the testicular tissue. Importantly, intratubular germ cell neoplasia, atrophy or lithiasis were not observed. Periodic Acid-Schiff (PAS) staining did not reveal any microbiological agent but highlighted the fact that the basal membrane of the seminiferous tubules was sometimes breached by the inflammatory infiltrate. Immunohistochemistry was performed and demonstrated that the inflammatory cells were composed of a majority of T cells (CD3 +, Figure 3 upper picture) mixed with some B cells (CD20 +, Figure 3 lower picture), few plasma cells (CD138 +) and very few histiocytes (CD68 +). Immunoreaction with the antibody anti-treponema was negative.

## Discussion

Testicles are usually considered as immunologically privileged organs, that is to say that lymphocyte activation is, under normal conditions absent [3]. Sertoli cells form a blood-testis barrier, physically isolating the testicle tissue from the systemic inflammatory cells. They also produce proteins inhibiting the proliferation of lymphocytes. Besides, Leydig cells produce testosterone which controls the proliferation of lymphocytes. Other cell types are hypothesized to play a role in the immunomodulated testicular environment, such as testicular macrophages or the germinal epithelial cells themselves. Under pathophysiological conditions, when spermatozoa leak from the tubules to the interstitium, inflammation can occur and classically leads to formation of spermatic granuloma [4]. Our main hypothesis was that this case of orchitis was caused by Crohn’s disease. We therefore listed the main possible etiologies of the focal mononuclear infiltrate in our patient, and evaluated the likelihood of each.

### Tumor

The most important differential diagnosis was a burnt-out/regressed germ cell tumor [5]. However, no scar, no intratubular germ cell neoplasia and no coarse intratubular calcifications were observed, which seem to be the most specific histological findings of a burnt-out germ cell tumor [5]. Less specific features of a regressed tumor were also absent, such as microlithiasis or testicular atrophy [5]. On the other hand, a lymphoma was excluded here given the lack of atypia of the infiltrating cells and their phenotype. The majority of lymphocytes were T cells as opposed to common testicular lymphoma which are usually B cell neoplasms, and there was very local involvement of the organ which is very uncommon in lymphoma where diffuse tumor infiltrate predominates [6].

### Orchitis associated with Crohn’s disease

Due to the inflammatory bowel disease in our patient, our first hypothesis was orchitis linked with Crohn’s disease. Crohn’s disease preferentially involves the gut, but numerous extra digestive manifestations have been described, such as the skin and mucosa, the joints, and the eyes [1]. They might be due to antigens shared by the gut and extradigestive organs. Lesions are usually composed of granulomas and multinucleated giant cells. The lymphocytic infiltrate of Crohn’s disease is usually composed of a polymorphous inflammatory infiltrate with more T cells than B cells [7]. In our case, there were strictly no giant cells or granulomas, but a majority of T cells. It must be noted that diagnosis of sarcoidosis was excluded because of the lack of granulomatous lesions [8].

### Infection

#### Viral infection

Mumps orchitis is considered as the archetype of viral lesion of the testis [9]. Braaten *et al.* listed a dozen cases with probable viral orchitis mimicking cancer, in which the tubules were never effaced by the inflammatory infiltrate, contrary to our case [10]. The majority of infiltrating lymphocytes are T cells, with only very few B cells, and this feature is present in our description [6]. Nevertheless, in our case, the kinetics of the clinical complaint argued against a viral cause since our patient reported onset of testicular pain several months prior to consulting and undergoing orchiectomy. In addition, neither hemorrhage nor neutrophils, which seem characteristic of a viral cause, were observed in the pathological territories.

#### Syphilis

Syphilis is another cause of testicular infection with mononuclear inflammatory infiltrate [11]. This hypothesis was rejected in our patient because of the small number of plasma cells, lack of vascular abnormalities and negative immunoreaction against spirochetes. Other infectious causes (tuberculosis, leprosy, fungal infection) were excluded given the nature of the inflammatory infiltrate and lack of microbial agents labeled on PAS staining [12].

### Vascular effects

The possibility of segmental testicular infarction was raised but appeared very unlikely since ultrasound examination revealed hypervascularization and microscope observation did not show any fibrosis, hyalinization, atrophy of the tubules, ghost outlines of tubules or any vascular abnormality.

### Traumatic effects

Acute trauma is highly unlikely because of the chronic evolution of the symptoms (several months) and absence of history of trauma reported by the patient. Intermittent torsion could be an alternative explanation. However, microscopic description lacked all the following features as compiled by Kao *et al.*, evoking such a cause, i.e. segmental haemorrhage, damaged blood vessels and ghost outlines of the tubules [13].

Given the lack of granuloma and a notion of traumatism, inflammation secondary to sperm extravasation was excluded [14].

### Iatrogenic effects

Iatrogenic damage is very unlikely. Not only no mention of such a phenomenon has been retrieved from the literature on similar cases in patients treated by infliximab or any other anti-TNF therapy, but on the contrary, non-lymphomatous mononuclear lymphocytic infiltrate should be attenuated by this immunosuppressive drug. In addition, the focality of the described lesion appears to contradict this hypothesis given that immunotherapy was systemic (intra-venous injections).

### Idiopathic effects

Interestingly, a similar case of lymphocytic orchitis leading to orchiectomy was reported previously and the authors concluded non specific or idiopathic orchitis [15]. However, contrary to our case, the inflammatory lesions were more diffuse and the pain was more acute with no reported history of autoimmune disease.

## Conclusion

Since all other plausible causes were rejected, we tend to think that this inflammatory lesion is a non granulomatous testicular manifestation of Crohn’s disease. To our knowledge, this would be the first report of non-granulomatous testicle related to Crohn’s disease in the medical literature.

## Consent

Written informed consent was obtained from the patient for publication of this Case Report and any accompanying images. A copy of the written consent is available for review by the Editor-in-Chief of this journal.
